# Epigenetic Genome Modifications during Pregnancy: The Impact of Essential Nutritional Supplements on DNA Methylation

**DOI:** 10.3390/nu16050678

**Published:** 2024-02-28

**Authors:** Maciej W. Socha, Wojciech Flis, Mateusz Wartęga

**Affiliations:** 1 Department of Perinatology, Gynecology and Gynecologic Oncology, Faculty of Health Sciences, Collegium Medicum in Bydgoszcz, Nicolaus Copernicus University, Łukasiewicza 1, 85-821 Bydgoszcz, Poland; wflis@copernicus.gda.pl; 2 Department of Obstetrics and Gynecology, St. Adalbert’s Hospital in Gdańsk, Copernicus Healthcare Entity, Jana Pawła II 50, 80-462 Gdańsk, Poland; 3 Department of Pathophysiology, Faculty of Pharmacy, Collegium Medicum in Bydgoszcz, Nicolaus Copernicus University, M. Curie-Skłodowskiej 9, 85-094 Bydgoszcz, Poland; mwartega@cm.umk.pl

**Keywords:** pregnancy, nutrition, epigenetics, DNA methylation, folic acid, choline

## Abstract

Pregnancy is an extremely stressful period in a pregnant woman’s life. Currently, women’s awareness of the proper course of pregnancy and its possible complications is constantly growing. Therefore, a significant percentage of women increasingly reach for various dietary supplements during gestation. Some of the most popular substances included in multi-ingredient supplements are folic acid and choline. Those substances are associated with positive effects on fetal intrauterine development and fewer possible pregnancy-associated complications. Recently, more and more attention has been paid to the impacts of specific environmental factors, such as diet, stress, physical activity, etc., on epigenetic modifications, understood as changes occurring in gene expression without the direct alteration of DNA sequences. Substances such as folic acid and choline may participate in epigenetic modifications by acting via a one-carbon cycle, leading to the methyl-group donor formation. Those nutrients may indirectly impact genome phenotype by influencing the process of DNA methylation. This review article presents the current state of knowledge on the use of folic acid and choline supplementation during pregnancy, taking into account their impacts on the maternal–fetal unit and possible pregnancy outcomes, and determining possible mechanisms of action, with particular emphasis on their possible impacts on epigenetic modifications.

## 1. Introduction

The preconception period and the period of pregnancy are special times in every woman’s life. Pregnancy involves many physiological adaptations that may influence a woman’s behavior and health. Moreover, pregnancy adaptations often require additional supplementation during pregnancy. In recent years, there has been a noticeable increase in women’s awareness of the use of supplementation, both in the preconception period and during gestation [1]. Moreover, a significant improvement in the awareness of women (and often their partners) regarding the overall course of pregnancy and possible pregnancy-associated health risks can be observed [2]. The basis for the increase in this awareness is probably the wide availability of the Internet and numerous educational programs. Additionally, an increasing percentage of physicians draw attention to the necessity and positive effects of supplementation during pregnancy, which further encourages patients to take such actions. Therefore, an increasing percentage of pregnant women try to follow the principles of a proper diet, perform appropriate exercises, and ingest supplementary preparations. According to recent studies, a significant number of the surveyed women reach for supplementary preparations during pregnancy [3,4,5].

During pregnancy, the demand for nutrients increases dramatically, including an increased demand for vitamins, choline, folic acid, etc., which ensure the proper course of pregnancy and maintain proper fetal development [6]. The levels of many nutritional factors in the blood serum decrease (or remain at low, insufficient levels) during pregnancy unless they are supplemented [6,7]. Referring to recent research, it can be seen that consuming insufficient nutritional supplements may be associated with a wide range of pregnancy complications, such as Down syndrome, neural tube defects, anemia, pregnancy-induced hypertension (and preeclampsia), fetal growth restriction, gestational diabetes, and an increased percentage of cesarean deliveries [8,9]. Additionally, improper nutrition during pregnancy may also have long-term effects on the newborn child, such as asthma, congenital heart defects, allergies, and neurodevelopmental disorders [1,2,8,9,10,11].

Molecules provided with a proper diet are crucial in supporting metabolic functionality and activity, including cell proliferation and differentiation and cell signaling, which regulate tissue function, growth, and homeostasis [12]. Substances such as vitamins (A, D, K, and group B vitamins), choline, folates, inositol, etc., are involved in several stages of fetal, maternal, and placental interaction, working to enable a healthy gestation period. Recently, the topic of epigenetics in the context of maternal–fetal medicine has been gaining more and more attention. Substances provided with the diet, apart from their obvious direct impacts on cellular metabolism and homeostasis, may have indirect effects through epigenetic modifications of the genome. These substances may participate in epigenetic modifications by acting as methyl-group donors. Nutrients specific to epigenetic pathways may directly and indirectly impact the epigenome by influencing the processes of DNA methylation and histone modification [13,14]. These are primarily folic acid and choline [14]. Those molecules play a crucial role, one which indirectly affects the entire cycle of one-carbon groups and the biosynthesis of nucleotides. Because epigenetic modulation is one of the elementary programming processes, it seems obvious that incorrect folate or choline supplementation may be correlated with abnormalities in fetal programming and an increased tendency to fetal disorders [12,13,14,15].

The aim of this review is to present the current state of knowledge regarding the impacts of crucial substances such as choline and folic acid on maternal–fetal medicine and determine their possible mechanisms of action, with particular emphasis on the impact on epigenetic mechanisms.

## 2. Epigenetics

**Figure 1:**
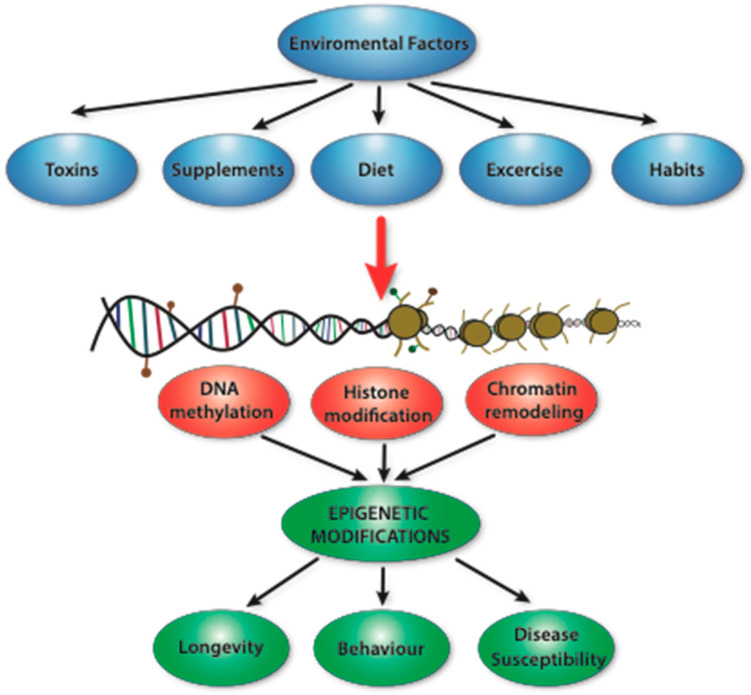
Diagram showing an overview of epigenetic mechanisms.

Currently, epigenetics refers to the changes occurring in gene expression that are not in direct correlation with changes in the DNA sequence itself [16]. The epigenetic mechanisms are responsible for specific gene expression without altering the genetic code. Environmental factors such as exposure to certain substances (provided with the diet), physical activity, or stressors can interact with genes and modulate their expression and function without altering the nucleotide sequence [17,18]. Epigenetic patterns may change throughout life—influenced by environmental exposures or nutritional status [18]. Changes in epigenetic patterns may lead to an alteration in the expression of specific genes, which can have downstream effects, such as changes in the risks of specific diseases, the metabolism, or behavioral patterns. Therefore, epigenetic signatures influenced by specific factors may determine human disease susceptibility, longevity, or, even, behavior [18,19,20] (Figure 1).

Epigenetic modifications involve DNA methylation, histone protein modification, or chromatin remodeling [21]. DNA methylation reaction is mediated by a family of DNA methyltransferases (DNMTs) and involves the addition of a methyl residue (-CH_3_ group) to the 5′ position of a cytosine, leading to 5-methylcytosine (5-mC) formation [22]. DNA methyltransferase exists in three forms (DNMT 1-3), with DNMT3 occurring in two isoforms—DNMT3a and DNMT3b. DNMT3a and DNMT3b methylate de novo DNA, while DNMT1 methylates the previously non-methylated hemi-DNA strand to generate a complementary DNA sequence [23]. Methylation occurs mainly within cytidine-guanine dinucleotide (CpG), which occurs repeatedly throughout the genome. The estimated range of CpG methylation is 70–80% throughout the DNA sequence in human cells [24]. Methylation of the CpG region located in the promoter of a specific gene increases the ability to bind other proteins to the methylated promoter region, leading to the blocking of the transcription of the specific gene [21,22]. Subsequently, the presence of methylated CpG dinucleotide residues prevents the transcriptional activity of specific genes that are expected to remain “silenced” in a cell-type-specific manner [25,26].

Epigenetic modification of histones is vastly more complex [27]. DNA double strand is densely packed into groups of approximately 146 base pairs, which are tangled around the proteins of histones. This complex with base pairs weaved around histones forms nucleosome, which is a subform of DNA packing [28]. From a chemical point of view, each nucleosome consists of the duplicated four core histones—H2A, H2B, H3, and H4. The entire nucleosome is covalently bonded by histone H1 [18].

There are several groups of enzymes that can modify histone proteins. Histone acetyltransferases (HATs) are enzymes that transfer the acetyl group onto the amino group of a lysine residue within the histone tail, leading to the positive charge of lysin neutralization [29]. In contrast, histone deacetylases (HDACs) work the opposite way—removing the acetyl residue restores the positive charge, which strengthens the connections of histones with the DNA [29,30]. Therefore, HATs can be classified as transcription activators, whereas HDACs are known as strong transcription suppressors. Another group of enzymes are histone phosphatases and kinases; these add or remove a phosphate residue from the hydroxyl group of the histone tail, leading to the removal or restoration of the positive charge, respectively. HATs and histone kinases are known as transcription activators, whereas HDACs and histone phosphatases are responsible for restraining transcriptional activity [22,23,24,25,26,27,28,29,30,31,32,33].

In addition to influencing the ionic charge of the protein (through acetylation and phosphorylation), epigenetic modification of histones can also take place through methylation [34]. Histone lysine methyltransferase (HKMT) and arginine methyltransferase (PRMT) are enzymes responsible for the methylation of histone tails, leading to gene transcription alteration [35]. Similarly to the process of DNA methylation, the addition of a methyl residue to the histone tail (by specific methyltransferases) leads to a change in the transcriptional activity of a specific region of the genome.

Regulation of gene transcription may also occur as a result of changes in the spatial conformation of chromatin [36]. A process called chromatin condensation allows a formation of a tightly packed, histone-rich structure to form separate chromosomes. The greatest compaction occurs during the metaphase of cell division [37]. Tight packaging of the genome leads to the production of heterochromatin, which becomes inaccessible to transcription factors, leading to the silencing of specific genes. In turn, loosening the chromatin structure results in the formation of euchromatin, which is available to transcription factors [38]. Chromatin remodeling, leading to gene silencing or activation, is under a strict control provided by an ATP-dependent protein complex or by histone-modifying proteins such as Polycomb-group proteins [21].

The ATP-dependent chromatin-remodeling protein complex, known as SWI/SNF, has catalytic activity in detaching specific histones from DNA, leading to the loosening of the chromatin structure. In turn, Polycomb proteins participate in chromatin condensation, leading to gene silencing. They produce protein multimers—protein repressive complexes (PRC), which, when bound to histones, lead to their mono-ubiquitination, which causes tighter packing of chromatin, leading to the inhibition of gene transcription and specific gene silencing [39,40,41].

DNA methylation seems to be not only the best-studied DNA modification pathway but also the most important mechanism of epigenetic genome modification [42]. The DNA methylation process cannot occur without molecules known as methyl-group donors. The main cellular donor of methyl groups is S-adenosylmethionine (SAM) [42]. Apart from playing a crucial role in DNA methylation, SAM is greatly involved in other cellular biochemical pathways, such as antioxidant formation, the synthesis of neurotransmitters, or phosphatidylcholine synthesis [43]. This molecule is synthesized during the “one-carbon metabolism”—a biochemical pathway responsible for the maintenance of cellular homeostasis, with additional participation of methyl-group donors [44]. These reactions support a vast group of interweaving metabolic pathways, such as synthesis of neurotransmitters, nucleotide metabolism, and/or lipids biosynthesis. The main biochemical pathways involved in a one-carbon cycle are methionine and folate cycles, among which the methyl-group donors such as choline, betaine, vitamins B6 and B12, and folate are crucial players [45].

In the cytosol, SAM is formed by adenylation of methionine catalyzed by methionine S-adenosyltransferase (MAT) [46,47]. Methionine is supplied through the diet or is de novo generated from homocysteine (hCy). After donating its CH_3_-group to a specific acceptor, SAM is transformed into S-adenosylhomocysteine (SAH), which is a strong metyltransferares competitive antagonist [48]. SAH is subsequently hydrolyzed to homocysteine and adenine. Adenine can subsequently enter into the purine-ring synthesis reaction [46,49]. In turn, homocysteine can be further transformed in several pathways. First, hCy can be transsulfurated, where it combines with serine to form cystathionine in a reaction catalyzed by cystathionine β-synthase (CBS) [50]. Secondly, hCy can convert to methionine, which will enhance further SAM production. Homocysteine can be converted to methionine via two biochemical pathways [47,50,51]. Betaine-homocysteine methyltransferase (BHMT) catalyzes the transfer of a methyl group from betaine (which is a metabolite of choline) to hCy, leading to the formation of methionine and dimethylglycine [52]. This pathway is particularly active in the muscles and liver, due to the high content of betaine and choline in these organs [47]. The second methionine regenerating pathway includes the folic acid pathway [53]. 5-methyltetrahydrofolate-homocysteine methyltransferase (MS) generates methionine by the transfer of the 5-methyltetrahydrofolate (5-mTHF) methyl moiety to hCy, leading to the methionine and tetrahydrofolate (THF) formation [54]. Both a decrease in the concentration of choline (and its metabolites) and folic acid lead to a significant increase in the concentration of homocysteine in the cell [46,54]. This suggests that methionine, folate, and choline metabolic pathways interweave and strongly interact at the point where hCy is metabolized to methionine. Transmethylation reactions occurring in the one-carbon cycle are strictly regulated by both external factors and the metabolites themselves. The ability to regenerate methionine from homocysteine in the folic acid pathway is strongly dependent on vitamin B12 availability, which is a cofactor of MS [46]. Overproduction of SAM causes inhibition of BHMT-dependent methionine regeneration [52]. Moreover, excess SAM inhibits methylenetetrahydrofolate reductase (MTHFR), leading to a decrease in the concentration of 5-mTHF, which participates in the regeneration of methionine [55]. Taking the above into consideration, it can be assumed that the cell’s ability to synthesize SAM is strictly dependent on the current availability of methionine, choline (and betaine), and 5-methyltetrahydrofolate (5-mTHF) [56]. Without ensuring an adequate supply of the substrates participating in the one-carbon pathway, it becomes impossible to generate an appropriate number of methyl-group donors (such as SAM), which are a link between cell metabolism and DNA methylation. Subsequently, this imbalance may translate into the impairment of the process of epigenetic modification of the genome [46,52,56].

Currently, much attention is given to the influence of external factors, such as dietary habits, on the development of the fetus from the moment of conception [57]. Moreover, it is believed that gestational (or pre-conceptional) exposure to environmental factors may epigenetically determine the phenotype or disease-susceptibility of the offspring [58]. After fertilization of the oocyte by the sperm, the DNA methylation pattern of the genome is erased, and then the de novo methylation process of the newly formed zygote occurs in order to have pluripotent cells [18,56]. During this process, a completely new DNA methylation pattern will be created, one which will be passed on to subsequent cells. DNA methylation is a key process for normal physiological development and one that plays an important role in gene expression during future cell differentiation. Additionally, during this time, female embryos will have their X chromosomes inactivated, leaving only one copy of each gene linked to the X chromosome to be expressed [18,57,58]. Taking all the above into consideration, we believe that the period of cellular differentiation occurring immediately after fertilization is the crucial moment in which proper DNA (and histones) methylation is able to ensure the proper, uninterrupted development of the fetus. Therefore, we believe that ensuring appropriate supplementation (leading to the proper supply of methyl-group donors) during this period is crucial for proper fetal development. For the rest of this review, we would like to focus on choline and folic acid and their possible roles in fetal development, emphasizing their possible impacts on epigenetic programming.

## 3. Choline

Choline, being a quaternary ammonium cation, is a water-soluble molecule that is crucial for proper homeostasis [59]. This micronutrient serves as the starting material for several important metabolites which are key players in fetal development. There is an increased demand for choline during pregnancy, especially in late gestation [60]. Referring to the research, it can be determined that pregnant women currently consume too little choline [60]. Insufficient choline ingestion can lead to serious pregnancy complications such as premature birth, preeclampsia, and fetal growth restriction (FGR) [59]. Therefore, a sufficient choline intake is pivotal for proper fetal development and homeostasis. An adequate intake level of 425 mg/day has been established for women, with up to 550 mg/day during pregnancy and lactation [1,61].

Choline (and its derivatives) are involved in a variety of processes, such as the biogenesis of cell membranes, cell divisions, DNA methylation, lipid transport, and myelination of nerve axons [49]. Choline can be supplied with food and can also be synthesized de novo [62]. The highest amounts of choline can be observed in red meat, eggs, and fish [62]. The endogenous synthesis of choline occurs mainly in the liver [62]. Endogenous choline is mostly obtained from phosphatidylethanolamine. Phosphatidylethanolamine N-methyltransferase (PEMT) sequentially methylates phosphatidylethanolamine to phosphatidylcholine using three molecules of SAM as a methyl donor [62,63]. Then, the newly formed phosphatidylcholine is enzymatically cleaved by phospholipases to choline or can participate in subsequent cell membrane biogenesis. Despite the consumption of three SAM molecules in the choline synthesis process, the concentration of S-adenosylmethionine is maintained via the BHMT pathway [64]. Interestingly, estrogen can upregulate PEMT expression in the human liver, leading to increased choline production during pregnancy [65,66]. This may suggest that the upregulation of endogenous choline de novo synthesis in a pregnant woman’s liver occurs during the gestation period.

A portion of choline is acetylated into acetylcholine (Ach) by the enzymatic activity of choline acetyltransferase (ChAT) [67,68]. Subsequently, acetylcholine is stored in neuronal membranes, where it acts as a neurotransmitter [69]. This is particularly pivotal in neurons with increased choline consumption, such as cholinergic autonomic neurons. The central nervous system cannot synthesize choline itself. It is provided by two main sources. The first is Ach degradation to free choline by acetylcholine esterase (AchE). The second pathway includes hydrolysis of phosphatidylcholine by specific phospholipases. Subsequently, free choline is stored in neuronal membranes for further Ach synthesis [69,70,71,72].

Choline is the precursor for several cell membrane phospholipids, such as phosphatidylethanolamine, sphingomyelin, and phosphatidylcholine. These choline-derived phospholipids are responsible for maintaining the structural and functional integrity of cellular membranes, including neuronal membranes [73]. They also participate in other physiological processes essential for normal brain development, such as signaling, cell proliferation, and myelination [73,74,75].

Apart from the obvious direct impacts of choline (and its derivatives) on specific cell metabolic pathways, it is also worthwhile to consider the indirect impacts of choline on epigenetic mechanisms. Recently, choline has emerged as a promising possible modifier of the genomic sequence that modulates gene expression by affecting the concentration of SAM, which is the main cellular methyl donor molecule participating in epigenetic mechanisms such as DNA and histone methylation [21]. As mentioned earlier, de novo choline synthesis consumes three SAM molecules. However, choline, through its indirect effects, is one of the factors that significantly increases the availability of SAM in the cell [45,63]. Recent studies have shown that choline contributes to the methylation of homocysteine, leading to a reduction in its concentration [76]. Moreover, oral choline supplementation was associated with a significant decrease in hCy concentrations [76]. We believe that this is possible mainly due to the choline metabolite—betaine. Betaine is a molecule containing three reactive methyl groups bonded with a nitrogen atom [77]. It is synthesized through choline oxidation via choline dehydrogenase and betaine aldehyde dehydrogenase [78]. One of three methyl groups previously associated with betaine is transferred to homocysteine, leading to methionine and dimethylglycine (DMG) formation in a reaction catalyzed by betaine homocysteine N-methyltransferase (BHMT) [78,79]. Then, methionine can transform into SAM, which will donate its methyl groups to a specific acceptor. DMG is subsequently partially eliminated through urine and partially metabolized into sarcosine and glycine [78,79,80].

As mentioned earlier, choline intake by pregnant women is largely insufficient. Studies have clearly shown that both supplementation and choline deficiency during various stages of human development have consequences on the brain function, neuronal activity, and cognitive functions of adult offspring [81]. Firstly, prenatal choline supplementation positively influenced the performance of memory-related tasks conducted later in the offspring’s life [82]. Moreover, a choline-deficient diet led to significant impairment in learning and memory skills in mice [83]. Additionally, intrauterine exposure of the developing fetus to choline has a positive effect on the proliferation of progenitor cells, while inhibiting apoptosis of hippocampal cells, leading to the improvement of hippocampal functioning [82,84,85,86]. Finally, choline supplementation positively impacted visuospatial and auditory memory in offspring [84,85,86]. It has also been shown that maternal choline supplementation during pregnancy can exert a neuroprotective effect and attenuate cognitive impairment associated with seizures [87,88,89,90]. Furthermore, it has been shown that neural tube defects (previously mainly associated with folic acid depletion) can be associated with choline supplementation [91]. According to the research, patients with low choline levels had a significantly increased risk of neural tube defects (NTD) [92,93,94,95].

It also turns out that choline supplementation can also alleviate symptoms related to pathologies occurring during pregnancy. Fetal alcohol exposure can lead to congenital fetal alcohol spectrum disorders (FASD), which are characterized by developmental disabilities such as congenital impairment, seizures, and poor growth [96]. Choline supplementation in offspring with FASD symptoms was associated with better verbal memory, fewer behavioral symptoms of attention deficit hyperactivity (ADHD), and increased non-verbal intelligence [97]. However, choline supply had no effect on the motion disorders occurring in this group of disorders [98].

The potential involvement of choline in autism spectrum disorder (ASD) has also been postulated [99]. This developmental disorder is characterized by difficulties in social communication and interaction, with additional restrictions and patterns apparent in social behavior. Referring to the research, these disorders may be caused by improper provision of nutrients during pregnancy [99,100]. Metabolic mapping of deep brain structures revealed that patients with ASD had significantly lower concentrations of choline in gray- and white-matter structures, compared with patients without developmental disorders [100,101]. Moreover, it was also shown that patients with ASD had significantly lower concentrations of choline (and its metabolites) in their blood plasma [102]. This may suggest that choline may be one of the crucial players ensuring proper brain development and may also prove to be an important biomarker of the severity of cognitive disorders.

It is hypothesized that perinatal choline supplementation may prevent the occurrence of cognitive disorders (such as ASD) or mental illnesses such as schizophrenia [103]. Expression of the *CHRNA7* gene and its receptor product, the α7-nicotinic receptor (among other receptors), are significantly reduced in specific disorders, especially in ASD or schizophrenia [103]. α7-nicotinic acetylcholine receptor is strongly involved in the maturation of GABA synapse inhibitors. Referring to the research, it has been noted that gestational choline supplementation was associated with the enhancement of the expression of the *CHRNA7* gene and its product [103,104]. The offspring of patients who ingested choline during pregnancy showed significantly less social withdrawal and significantly fewer attention problems, compared with the placebo group [104]. We believe that choline supplementation can result in more effective maturation of GABA receptors, which may reduce excessive synaptic activation in the future and thus lead to a reduced risk of behavioral disorders or mental illnesses.

Undoubtedly, there is a connection between choline supplementation and proper brain development. Possible explanations for this phenomenon seem to be multifactorial. Firstly, phospholipids (which are derivatives of choline) are an essential component of the emerging cell membranes—their presence is crucial during the period of rapid cell division and myelination of newly divided neurons. Secondly, acetylcholine ensures the proper development of brain progenitor cells and enables proper cholinergic conduction, which is crucial in the developing brain. Third, as mentioned earlier, choline can directly influence growth stimulation while inhibiting apoptosis of deep brain structures such as the hippocampus. Finally, choline may exert an indirect effect on brain development through epigenetic mechanisms as a methyl-group donor. Studies have shown that a low-choline diet during pregnancy was associated with significant fetal decline of hippocampal DNA methylation of the *Cdkn3* (cyclin-dependent kinase inhibitor 3) gene and increased expression of its protein product, kinase-associated phosphatase (Kap), a cell cycle regulator that inhibits cell proliferation [105].

The effects of choline supplementation on the symptoms associated with genetic neurological disorders such as Down syndrome were also investigated. A rodent model of Down syndrome (DS) showed great depletion of cholinergic neurons of the basal forebrain, leading to impairment in cognitive functions, memory, and attention functions—symptoms often presented in Alzheimer’s disease [63]. Interestingly, maternal perinatal choline ingestion in the mentioned rodent model significantly improved cognitive functions in the offspring with DS [106,107]. Moreover, choline supplementation seems to not only have a positive effect on alleviating the cognitive symptoms of Down syndrome, but also may help reduce the risk of its occurrence. Referring to the conducted research, women who delivered a child with DS had significantly lower folic acid and choline concentrations [108]. Additionally, these patients had significantly higher homocysteine concentrations, compared to women with a child without cognitive disorders [108]. These data are extremely interesting for several reasons. First, these data show promising possible treatment options for alleviating Down syndrome symptoms in the offspring. Secondly, they indicate the interdependence between the metabolisms of folic acid and choline and confirm the fact that the biochemical pathways of the one-carbon cycle are interconnected and interwoven. Finally, these data may suggest that the appropriate concentration of methyl groups may translate into the risk of DS. As mentioned earlier, both choline and folic acid participate in the one-carbon pathway, which (among other things) is responsible for generating methyl residue donors. In cases of an insufficient supply of folic acid and choline, methionine synthesis decreases with an increase in hCy concentration. Improper methionine concentration translates into insufficient SAM production, which causes a decrease in the number of available methyl groups. As a result, hypomethylation of the kinetochore may occur—the latter is a protein connecting the centromere with the karyokinetic spindle fibers and determines the proper movement of chromosomes in the metaphase and anaphase of cell division [109]. Insufficient hypomethylation of the kinetochore impairs its function, which may lead to abnormal cell division, leading to the development of Down syndrome. Taking all of the above into consideration, we believe that choline (being strongly involved in the production of SAM) may significantly contribute to the reduction of cognitive symptoms occurring in a number of neurodevelopmental diseases and may also prove to be a factor that will have a positive impact in reducing the risk of Down syndrome in offspring. However, this topic requires further thorough research.

In addition to the described impact on fetal development, choline may also contribute to the proper course of pregnancy itself, which may translate into a reduction in the rate of pregnancy complications. Choline supplementation in the third trimester of pregnancy was associated with a significant decrease in the concentration and expression of Fms-like tyrosine kinase-1 factors (sFlt-1), which are the main antiangiogenic factors involved in the preeclampsia pathogenetic pathway [110]. Additionally, choline supplementation has been linked with positive modulation of oxidative stress, apoptosis, and inflammatory response in human placental trophoblast cells [111,112]. Both overexpression of sFlt-1 and inflammatory response (with the assistance of oxidative stress) occur within the preeclampsia pathogenetic pathway. We believe that choline, by significantly reducing the concentration of sFlt-1 and inhibiting the development of the inflammatory reaction, may contribute to the inhibition of the development of preeclampsia.

It is also believed that choline may positively affect fetal growth [113]. Referring to the research, rodents with an insufficient placenta, after choline supplementation, showed higher betaine concentrations, which, in turn, positively correlated with fetal weight gain in early pregnancy [114,115]. It is believed that choline may exert its effect on fetal growth via the insulin-like growth factor (IGF) axis [113]. Choline supplementation, by influencing DNA methylation and increasing gene expression, is associated with increased expression of specific IGF proteins, which leads to improved fetal growth [116].

Choline supplementation may also affect the hypothalamic–pituitary–adrenal (HPA) fetal axis. Excessive stimulation of the HPA axis during pregnancy may translate into increased risks of preeclampsia and fetal growth restriction [117]. Moreover, increased activity of the HPA axis (caused, for example, by the use of glucocorticosteroids or maternal stress) may lead to increased susceptibility to the development of hypertension and diabetes as an adult [118]. Apart from the negative impact of HPA axis stimulation on somatic disorders, it is believed that HPA hyperstimulation may be connected with psychiatric disorders and long-term neurobehavioral outcomes [119,120,121]. The latest research has shown that maternal choline supplementation led to the alteration (via the donation of methyl groups) of the methylation of genes, such as corticotropin-releasing hormone gene (*Crh*) and glucocorticoid gene (*Nr3c1*), in the placental tissue [122]. Subsequently, these changes led to a decrease in the cortisol concentration in the umbilical cord blood [122]. The above data show that choline prenatal supplementation may be a potent novel therapeutical option in pregnancies where excessive maternal stress leads to HPA axis hyperactivity. However, this interesting topic requires further thorough research.

Taking all the above into consideration, we believe that choline can exert positive effects on placentation and trophoblast cells by reducing the inflammatory response, reducing the expression of anti-angiogenic factors, and increasing the secretion of specific growth factors. These data clearly show that choline intake may be beneficial for pregnancies complicated by placental insufficiencies such as preeclampsia or fetal growth restriction. Overall, given the compelling data and encouraging results from the above studies, further examination of the relationships between maternal choline intake and placental function, fetal brain development, and future cognitive function is required.

## 4. Folic Acid

The term “folates” is a general term encompassing folic acid and its derivative compounds, such as dihydro, tetrahydro, and methyl, having specific metabolic activities in the cell [123]. Natural sources of folates include fruits, meat, and green, leafy vegetables [123]. Additionally, folic acid (the synthetic form of folates) is used as a fortifying factor in dietary supplements [124].

**Figure 2:**
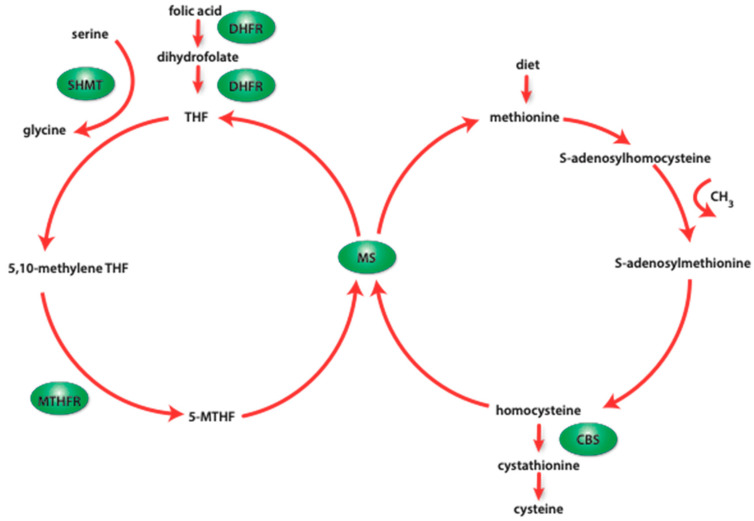
Diagram showing the course of the one-carbon cycle. MTHFR—methylenetetrahydrofolate reductase; DHFR—dihydrofolate reductase; MS—methionine synthase; SHMT—serine hydroxymethyltransferase; CBS—cystathionine β-synthase; 5-MTHF—5-methyltetrahydrofolate.

Folic acid (as well as its metabolites), in addition to its effective direct impact on a wide range of metabolic processes occurring in the cell, is also a well-known epigenetic modifier. Apart from participating in the synthesis of the purine ring, it also indirectly participates in the donation of methyl groups in epigenetic transformations [125]. After enzymatic cleavage of ingested nutrients in the intestine, folic acid enters the cells (mainly the liver, kidney, and skeletal muscles), where it is reduced to dihydrofolate (DHF), and then to tetrahydrofolate (THF) by dihydrofolate reductase (DHFR) [125]. Subsequently, serine hydroxymethyltransferase (SHMT) catalyzes the reaction of conversion of THF to 5,10-methylenetetrahydrofolate (5,10-mTHF). This reaction requires serine, glycine, and vitamin B6, as coenzymes, to occur properly. Then, 5,10-mTHF is reduced to 5-mTHF in a reaction catalyzed by methylenetetrahydrofolate reductase (MTHFR) [126]. In this metabolic form, folic acid enters the one-carbon cycle, where it actively participates in the generation of methyl residue donors. THF and 5-mTHF are among the most active metabolites of folic acid [125,126,127]. As mentioned earlier, the one-carbon cycle consists of intersecting biochemical pathways (Figure 2). Generally, these pathways can be divided into folate-dependent and folate-independent pathways. The folate-independent pathway consists of a series of reactions involving choline and its metabolites, whereas the folate-dependent pathway consists of a series of reactions involving hCy, 5-mTHF, and vitamin B12 [127,128]. If the correct concentrations of substrates and cofactors are ensured, methionine synthase (MS) transfers the methyl group from 5-methyltetrahydrofolate to homocysteine, leading to the formation of methionine and tetrahydrofolate [129]. Then, methionine can be converted into SAM, which will serve as a methyl-group donor [129]. In turn, the resulting THF can be converted back to 5,10-meTHF in a reaction catalyzed by SHMT [130]. There is no doubt that the entire one-carbon cycle is subject to strong dependencies resulting from the network of regulatory factors as well as the availability of substrates. The most important regulator of the folic acid-dependent side is undoubtedly vitamin B12, which is a cofactor of methionine synthase [131]. When vitamin B12 is deficient in a cell, a phenomenon called the “folate trap” occurs [126,131]. Vitamin B12 deficiency ensures the proper functioning of methionine synthase, leading to the accumulation of 5-mTHF and the inability to metabolize it to THF. Moreover, the inactivity of this part of the one-carbon cycle pathway leads to the accumulation of hCy and a significant deficiency of SAM. A small percentage of hCy can be converted to methionine in the betaine-dependent pathway, but this route is insufficient for ensuring the adequate efficiency of methionine production [127,128,129]. The accumulating excess hCy can be transsulfurated to cystathionine in a CBS-catalyzed reaction. However, it is worth paying attention to the presence of possible molecular relationships. As it turns out, SAM has the ability to allosterically activate the CBS enzyme, which drives the transsulfuration reaction with an excess of SAM and hCy [129,130,131,132]. Taking the above into account, we believe that SAM deficiency (resulting from vitamin B12 deficiency) may lead to inhibition of CBS activity, which will ultimately lead to a decrease in hCy transsulfuration. This, in turn, will cause the accumulation of homocysteine, which will be excreted into the bloodstream. Moreover, under the influence of CBS, homocysteine is transformed into cysteine, which is the precursor of glutathione [133]. Glutathione (γ-glutamyl-cysteinyl-glycine) is the main participant in redox processes in the body [133]. Excessive concentration of hCy in the bloodstream increases its susceptibility to its oxidation, which may lead to an increase in the production of reactive oxygen species (ROS) [133]. Summing up, it is plausible that vitamin B12 deficiency may result in a decrease in SAM concentration, which will translate into inhibition of CBS activity and, as a result, may lead to a reduction in glutathione synthesis, which will affect cell redox reactions. Bearing the above in mind, we believe that improper functioning of folic acid metabolism may translate not only into a reduction in the available pool of methyl moiety donors but may also exert more long-term effects in the form of impairment of cellular redox reactions.

Folic acid appears to be crucial for the well-being of pregnancy. The demand for this nutrient increases significantly during gestation [134]. Apart from the obvious contribution to DNA synthesis processes (direct participation in the synthesis of the purine ring) and amino acid metabolism, folates also contribute to epigenetic processes [6,135]. Their role in ensuring the proper course of pregnancy applies to almost all stages of gestation [6,56].

The relationship between folic acid deficiency and neural tube defects is well established. Numerous studies indicate that folic acid deficiency during embryogenesis significantly affects the occurrence of neural tube defects (NTDs) [6,133,136,137,138,139,140,141]. There are also many reasons to believe that folic acid influences the proper closing of the neural tube through epigenic processes such as DNA methylation. First, the use of methionine cycle inhibitors or DNA methyltransferase mutations in animal models has led to the occurrence of NTDs [142]. Moreover, it has been shown that polymorphisms in genes encoding enzymes involved in the one-carbon cycle may contribute to an increased rate of NTDs [143]. MTHFR polymorphisms C677T and A1298C are associated with a greater risk of NTDs [144,145]. Additionally, the C677T MTHFR polymorphism has been linked with decreased levels of plasma folate and increased levels of hCy, as well as diminished DNA methylation [145,146,147,148,149,150]. These data clearly indicate the significant involvement of the folate-dependent pathway of the one-carbon cycle in the process of proper neural tube closing (NTC). We believe that by contribution to the methyl-group donor metabolism, folates can influence DNA methylation, which translates into the silencing/expressing of specific genes that regulate the proper course of cell migration during NTC. However, we suggest that this process should not be considered only with respect to the relation between the folate component and NTC. The relationships between all pathways of the one-carbon cycle have been demonstrated—those pathways are closely interdependent. Therefore, we believe that the remaining components of this cycle (such as choline, SAM, betaine, etc.), through their regulatory influence on folate metabolism, may also significantly influence NTC processes. However, this topic requires further thorough research.

Folic acid also appears to be pivotal for numerous physiological and metabolic pathways occurring during gestation. Referring to the research, it can be seen that folic acid takes an active part in the placentation process [125]. The placentation process mainly involves the invasion of uterine tissue by fetal trophoblast cells. Trophoblast cells penetrating the uterine spiral arteries cause their thorough reconstruction—converting them into high-capacity, low-resistant vessels, and allowing them to transport large volumes of blood [151]. The entire process consists of degradation and remodeling of the extracellular matrix (ECM), and is mediated by metalloproteinases (MMPs), accompanied by increased vasculogenesis and angiogenesis [152]. Additionally, ECM remodeling by penetrating trophoblast cells often leads to the generation of oxidative stress [153]. The research clearly shows the positive effects of folates on the placenta during early gestation. It was shown that the incubation of trophoblast tissues with folic acid solution resulted in a significant increase in trophoblast invasion, with simultaneous stimulation of MMPs secretion, which actively participates in ECM remodeling during trophoblast invasion [154]. Additionally, an increased vascular density in placental tissue was observed, which clearly indicated the intensification of angiogenesis processes [154]. Moreover, it has also been shown that folic acid deficiency may be associated with placental pathologies such as placental abruption [155,156]. Finally, low folic acid concentrations have been found in patients with preeclampsia [157]. In the context of preeclampsia, recent research indicates that folic acid supplementation may help reduce the incidence of this prenatal pathology [158]. Interestingly, folic acid supplementation has also been shown to be associated with a reduction in fetal growth restriction (FGR) occurrence [159].

We refer to pathologies such as FGR, PE, and placental abruption because these pregnancy complications mainly result from an incorrect placentation process. We draw attention to this phenomenon because it appears that folic acid may not only positively affect the placentation process per se but also could be of benefit in preventing prenatal pathologies associated with inappropriate placentation processes. Due to its multifactorial action, it is possible, not only to ensure the proper course of the placenta formation process, but also to ensure balanced, uncomplicated intrauterine fetal development.

In addition to the described impacts of folates on the proper course of gestation, it is also worth paying attention to the potential neurodevelopmental benefits of their supplementation. Animal studies have shown that folic acid deficiency during neurogenesis resulted in impaired cell migration and synaptogenesis, and a general reduction in the number of cerebellar and hippocampal neurons [160,161]. Additionally, an insufficient supply of folate during the preconception and pregnancy period is associated with an increased risk of ASD and ADHD in offspring [162,163,164]. Moreover, increased folic acid intake during pregnancy reduced the occurrence of neurodevelopmental disorders [164,165,166]. A possible explanation for these effects of folate on brain development and the risk of certain neurodevelopmental diseases may lie in epigenetic modifications, especially in DNA methylation pathways. Numerous studies indicate the involvement of folates in epigenetic regulations, mainly including DNA methylation [167,168,169]. It was shown that the maternal concentration of folic acid is proportional to the global methylation of the offspring’s genome—a high concentration of folic acid in the mother’s body correlated positively with DNA hypermethylation of the offspring’s genome [125,170,171]. Moreover, conducted research showed that patients with autism spectrum disorder had lower methionine concentrations and a significant decrease in the SAM to SAH ratio, with a simultaneous increase in homocysteine concentration [172,173,174,175,176]. Taking all of the above into consideration, we believe that folic acid deficiency (which actively participates in the metabolism of methyl donors) may translate into insufficient production of methyl donors, which may lead to alterations in specific DNA methylation patterns. This, in turn, may silence some genes and may ultimately lead to some neurodevelopmental diseases [177]. However, taking into account the multifactorial and polygenic pathogenetic nature of neurodevelopmental and cognitive disorders, we believe that this topic requires further, extensive research.

Summarizing the analyzed data, we believe that folic acid (and its metabolites) can have a significant positive impact on the course of pregnancy; not only directly, but also indirectly, through the influence on DNA methylation, they can condition the proper, undisturbed development of offspring.

## 5. Discussion

Apart from the obvious direct influences of substances such as folic acid and choline on proper gestation, it seems that those molecules are associated with additional indirect effects that may be determinative in the future life of the offspring. The aim of this review was to present the current state of knowledge regarding the impact of crucial substances such as choline and folic acid on maternal–fetal medicine, with particular emphasis on the impact on epigenetic mechanisms.

The presented data clearly show how important it is to ensure an adequate supply of supplements during pregnancy. Providing the correct intake applies not only to folic acid and choline but also to vitamins (such as vitamins B1, B12, and D3), inositol, and docosahexaenoic acid (DHA). Currently, more and more global medical societies recommend consuming a wide range of dietary supplements throughout pregnancy [178,179,180,181,182,183] (Table 1).

Compared to recent years, patients’ awareness of the importance of ensuring the correct concentration of supplements during the preconception period and gestation has increased significantly. Several global organizations have launched extensive awareness-raising campaigns aimed at increasing dietary supplementation during pregnancy. However, despite widely used information campaigns, it seems that the use of additional supplementation and patients’ knowledge in this area are still insufficient. Referring to the research, it can be seen that, in developed countries, the current consumption of supplements, especially folic acid and choline, is still not satisfactory [6,184,185,186,187]. Therefore, we deeply believe that it is extremely important to constantly strive to increase public awareness of the principles of proper nutrition and supplementation during pregnancy. With this in mind, we believe this approach will further reduce possible maternal and fetal complications resulting from the deficiency of key factors such as folic acid or choline. Apart from the presented data on the impact of selected dietary factors on specific genetic mechanisms determining the proper development of the fetus, it seems that the use of specific dietary patterns and maintaining appropriate diet may be associated with positive pregnancy outcomes. According to research, eating a diet rich in vegetables, fruit, whole grains, nuts, legumes, and seafood, and low in red, processed meat and fried foods is associated with a reduced risk of premature births [188]. Moreover, following such a diet may be associated with higher birth weights in newborns [188]. With the above in mind, we deeply believe that a specific, broadly held understanding of following a healthy diet may be associated with achieving better obstetric outcomes and reducing potential pregnancy and neonatal complications.

As mentioned earlier, supplementation deficiencies associated with various substances may translate into abnormal fetal development. Among other supplements, folic acid and iron deficiencies in a pregnant patient’s diet may be related to the occurrence of congenital heart defects (CHDs) [189,190,191]. Congenital heart diseases, caused by abnormal development of the heart during pregnancy, are among the most common birth defects worldwide [191]. Without the implementation of appropriate supplementation during pregnancy and proper diagnostics, CHDs lead to disastrous consequences for the newborn child. We would like to emphasize the importance of ensuring proper supplementation during pregnancy in order to minimize the risks of these dangerous diseases. However, it appears that, when a CHD is suspected, fetal echocardiography may prove to be a helpful diagnostic tool. Referring to the research, it can be seen that fetal echocardiography is a diagnostic tool with a high percentage of effectiveness in the diagnosis of heart defects [192,193]. With the above in mind, we believe that ensuring proper supplementation during pregnancy is an extremely important factor in reducing the risk of congenital heart defects. Moreover, we also believe that if supplementation deficiencies are detected during pregnancy, it is extremely important to perform fetal echocardiography, which will enable early detection of potential congenital heart defects. Thanks to this, it will be possible to provide the patient with appropriate, specialized care and implement appropriate procedures that will help reduce possible consequences for the newborn.

We would also like to draw attention to possible complications related to excessive use of individual supplements during pregnancy. Despite the well-known positive impact of folic acid supplementation on the course of pregnancy, it is worth noting that it may also have a negative impact. Referring also to large-scale campaigns to increase patients’ awareness of folic acid supplementation, concerns have arisen about excessive folic acid concentration in the entire population. Several studies show that patients can consume too much folate, which may have adverse effects [125,194,195]. Excessive folate intake during pregnancy may be positively correlated with an increased rate of allergic outcomes in offspring. Increased folate concentration was associated, in offspring, with food allergies, atopic conditions, and an increased risk of developing bronchial asthma [196,197]. A possible explanation for this phenomenon is the impact of increased folate supply on changes in the DNA methylation pattern, which may translate into the revealing of the phenotypes responsible for the development of allergies [197,198,199]. Moreover, folate oversupply may be associated with hepatotoxic effects on the maternal liver [200,201]. Nevertheless, the positive effect of folates on the proper development of the fetus and the proper course of pregnancy leaves no doubt. It is possible that the negative effects of folate supplementation result from their incorrect dosage or insufficient knowledge of patients in this area. In many countries, folic acid supplements are sold without a prescription. Therefore, many patients use them without prior consultation with a physician and are unaware of the correct regimen for taking these supplements. Therefore, we emphasize how important it is to constantly increase patients’ awareness of developing proper supplementation habits and to develop top-down dosage regimens for specific pregnancy supplements.

There are many reasons to believe that the components of the one-carbon cycle pathways (folate and choline) responsible for generating methyl-group donors are intertwined and participate in ensuring the proper rotation of the cycle. A recent study showed that pregnant women with low plasma folate had lower plasma betaine and higher DMG and tHcy, as compared to women with higher plasma folate levels [202]. Additionally, in patients with folic acid deficiency, the plasma levels of methionine and betaine remain unchanged, and the DMG concentration increases, which suggests that betaine, acting as a methyl-group donor, is able to compensate for folic acid deficiencies and ensure the proper functioning of the one-carbon cycle [202,203]. Taking the above into account, we believe that the substances involved in the metabolic pathways of the one-carbon cycle are strongly interconnected. The deficiency of one of the factors can be compensated for by increases in the activity and concentrations of the remaining components of the pathway, leading to the sustaining of the generation of methyl residues and the utilization of hCy at an appropriate level. This may suggest the presence of regulatory mechanisms operating on the basis of negative/positive feedback.

When considering the interdependence of methyl donors, we should not forget the joint impact these substances have on cells. Large amounts of choline are delivered to the fetal brain tissue across the placenta [204]. Interestingly, the placenta is one of the few organs capable of storing large amounts of choline (and its metabolites) [204]. It is highly possible that a high concentration of choline acts as a reserve, ensuring proper choline delivery to the fetal cells. Furthermore, increased choline concentration is associated with increased S-adenosylmethionine concentrations [71]. A high concentration of choline causes increased production of SAM, suggesting that the high concentration of choline in the fetal brain tissue is intended to provide large amounts of methyl residues, which directly translates into epigenetic modifications of DNA. Increased choline concentration (especially in brain tissue) undoubtedly contributes to the neurodevelopment of the central nervous system and neurogenesis. By influencing DNA methylation, choline ensures proper brain development, which translates into a reduced rate of neurocognitive and psychiatric disorders in offspring. Folic acid has similar effects—from a positive effect on the closing of the neural tube through proper placentation, to reducing the risk of behavioral disorders. The existence of the multiple mechanisms which ensure the multifactoral availability of choline and folic acid to the fetus suggests their great role in proper neurodevelopment. Moreover, it turns out that folic acid and choline can act synergistically. It has been suggested that the entirety of the one-carbon metabolism could be strongly involved in the proper neurodevelopment process. Referring to recent studies, concurrent intake of methyl donors (such as folic acid and choline) reduces the risk of NTDs to a greater extent, compared to taking only single substances such as folic acid [71,204]. This suggests that the intake of folic acid can be supplemented with the intake of choline, which may translate into a reduced risk of NTDs. This suggests that concurrent intake of several methyl donors may have a greater positive effect in terms of epigenetic modifications. This shows a new potential direction of research which may help reduce the risk of potential maternal–fetal complications. Moreover, it seems that the mentioned substances, through cooperation and mutual synergism in relation to epigenetic modifications, create a specific “DNA protection formula”, thanks to which it is possible to implement correct DNA methylation patterns; this translates not only into the undisturbed intrauterine development of the fetus, but also the future life of the offspring. However, considering that most of the research has been conducted on animal models, we believe this interesting topic requires further exploration.

Despite our great understanding of the cellular and molecular events that occur during fetal development, the search for the factors that modulate those pathways continues. A comprehensive understanding of the impacts of specific substances supplemented during pregnancy seems to be pivotal in the context of maternal–fetal medicine. In-depth knowledge in this field will enable us to provide means that can protect the woman and her unborn child from potential complications.

## Figures and Tables

**Table 1:** Table summarizing the recommendations of various medical societies regarding the supplementation of selected nutrients during pregnancy. DHA: docosahexaenoic acid.

Dietary Supplement	American College of Obstetricians and Gynecologists	National Institute for Health and Care Excellence/Royal College of Obstetricians and Gynecologists (NICE/RJOG)	The Polish Society of Gynecologists and Obstetricians
Folic acid	0.4 mg–0.6 mg/day	0.4 mg	0.4 mg–5 mg (depending on pregnancy complications)
Iodine	200 μg/day	Not applicable	150–200 μg/day
Vitamin D	1000–2000 IU/day	1000 IU/day	1500–2000 IU/day
DHA	Not applicable	Not applicable	600 mg/day
Choline	450 mg/day	400 mg/day	Not applicable
